# Patient-Reported Outcome Measures on Oral Hygiene, Periodontal Health, and Treatment Satisfaction of Orthodontic Retention Patients up to Ten Years after Treatment—A Cross-Sectional Study

**DOI:** 10.3390/ijerph19084843

**Published:** 2022-04-15

**Authors:** Barbro Fostad Salvesen, Jostein Grytten, Gunnar Rongen, Vaska Vandevska-Radunovic

**Affiliations:** 1 Department of Orthodontics, Institute of Clinical Dentistry, University of Oslo, 0317 Oslo, Norway; vaska.vandevska-radunovic@odont.uio.no; 2 Department of Community Dentistry, University of Oslo, 0317 Oslo, Norway; josteing@odont.uio.no (J.G.); gunnar.rongen@odont.uio.no (G.R.); 3 Department of Obstetrics and Gynecology, Institute of Clinical Medicine, Akershus University Hospital, 1478 Lørenskog, Norway

**Keywords:** long-term retention, fixed orthodontic retainers, periodontal health, treatment and outcome satisfaction

## Abstract

Background: This cross-sectional study evaluated patient-reported outcome measures (PROMs) on (1) oral hygiene, (2) periodontal health, (3) retainer failure, (4) orthodontic treatment satisfaction, and (5) outcome satisfaction in orthodontic retention patients. The purpose of the study was to evaluate whether orthodontic retention treatment is associated with patient-reported outcome measures on oral hygiene, periodontal health, and treatment satisfaction. Methods: A ten-item questionnaire on the five PROMs was conducted among 211 consecutive retention patients up to ten years following orthodontic treatment. Linear regression models were computed to detect possible associations between the PROMs and retention treatment or patient characteristics. Results: The presence of a fixed lingual retainer was not associated with the reduced ability to perform oral hygiene, self-perceived periodontal health, or orthodontic outcome satisfaction. Older patients were more content with the orthodontic treatment result (*p* < 0.05). Patients with fixed lingual retainers in the mandible were less satisfied with the course of orthodontic treatment (*p* < 0.05). Smokers more often reported gingival bleeding (*p* < 0.05). Females reported increased gingival recessions (*p* < 0.05) and perceived their teeth as longer than before treatment (*p* < 0.05). Longer orthodontic treatment duration corresponded to retainer failure (*p* < 0.05). Conclusions: In general, long-term orthodontic retention patients were satisfied with orthodontic treatment. These patients reported the satisfactory ability to perform adequate oral hygiene and periodontal health, and they communicated a high degree of treatment and outcome contentment. However, patients with a retainer in the mandible were less satisfied with orthodontic treatment.

## 1. Introduction

Successful orthodontic treatment relies on preserving good oral health and an aesthetic, functional, and stable treatment outcome [1,2]. Active orthodontic treatment is usually followed by a long retention phase in an attempt to maintain teeth in their corrected positions and to avoid unwanted long-term age changes [3]. For that purpose, most orthodontists use fixed bonded retainers, which have become increasingly popular and are better accepted by patients [4,5,6,7].

Both the orthodontic treatment phase and the orthodontic retention phase can potentially compromise oral hygiene due to plaque- and food-retentive appliances [8]. Thus, a significant concern for the use of long-term fixed retainers is their potential effect on periodontal health [9,10,11]. However, a literature review shows no clear consensus whether the use of long-term fixed retainers leads to the development of periodontitis [9,10,12,13,14].

The use of patient-reported outcome measures (PROMs) is intended to improve the aspect of health care that matters the most to patients themselves: their own subjective experience of treatment and outcome. Therefore, it is essential to evaluate patients’ reported symptoms and signs during and after orthodontic treatment. However, research on PROMs regarding oral health is lacking [15,16]. Whereas the main body of research has focused on objective outcomes measured by the clinician, few studies have explored the patients’ perspective on oral health using PROMs [16]. Moreover, patient satisfaction has generally received limited coverage in the orthodontic literature [17]. Patient satisfaction with orthodontic treatment has varied considerably in previous studies, and only a few studies have explored possible governing factors of patient satisfaction [18,19,20]. Similarly, long-term retention following active orthodontic treatment may influence self-perceived periodontal health and patient satisfaction. Yet, to our best knowledge, no studies have investigated whether self-perceived periodontal health, satisfaction with orthodontic treatment, and satisfaction with orthodontic treatment outcomes become affected in long-term orthodontic retention patients.

The current study was aimed to evaluate PROMs on oral hygiene performance, periodontal health, retainer failure, and orthodontic treatment and outcome satisfaction in orthodontic retention patients with and without retainers.

## 2. Materials and Methods

The study was approved by The Regional Committee for Medical and Health Research Ethics (2015/695), and it was conducted according to the declaration of Helsinki. Written informed consent was obtained from all patients.

### 2.1. Study Population

The current study was conducted on the same patient population as was explored in a previous report by the research group [21]. In brief, the study comprised patients who underwent routine retention control between three and ten years after orthodontic treatment at the Department of Orthodontics, University of Oslo. Between October 2015 and June 2017, 216 consecutive patients were invited to participate in the study. Two hundred eleven patients (115 females and 96 males) consented to inclusion. In comparison, five patients declined participation due to time constraints and one participant failed to fill out the majority of the questionnaire and was excluded.

Inclusion criteria were: (1) previous orthodontic treatment with fixed appliances on the buccal tooth surface and (2) orthodontic treatment started before 18.

Exclusion criteria were: (1) missing or extracted upper or lower incisors or canines, (2) patients undergoing/treated with orthognathic surgery, (3) craniofacial syndromes, and (4) cleft lip and/or palate.

All retainers considered in this study were fixed metallic retainers bonded with composite to the lingual/palatal aspect of the six anterior teeth. To a lesser extent, only canines in the mandible were bonded. Due to the study’s retrospective nature and an inconsistent bonding protocol with more than one specific retainer material, retainers were evaluated as one group.

### 2.2. Study Outcomes

The patients responded to a validated ten-item questionnaire about self-reported oral health, retainer failure, and orthodontic treatment and outcome satisfaction. All questionnaire items were binary (yes/no) and originally formulated in Norwegian, here presented in an English translation: (1) Do you think it is challenging to maintain a good hygiene regime in the anterior part of your dentition? (2) Do you experience any sensitivity in the anterior parts of your dentition? (3) Do your gums bleed after brushing your teeth, using dental floss or dental sticks? (4) Do you feel like your gums have retracted? (5) Do you feel like your teeth look longer? (6) Have you experienced any loosening of the retainer at any point? (7) Were you content with the treatment you received? (8) Are you content with the result/outcome of the treatment? The questionnaire also yielded information about (9) age and (10) gender. Lifestyle factors (e.g., smoking and use of snuff) and orthodontic treatment-related details (e.g., number of tooth extractions, duration of orthodontic treatment in months, and years since debonding) were obtained from the patients’ journal. A clinical examination revealed the presence or absence of retainers in the anterior aspects of the maxilla and the mandible.

### 2.3. Statistical Analysis

Sample size calculation was performed before study onset using SPSS 25.0.0.1 (IBM Corporation, Armonk, NY, USA). The necessary sample size was 200 when using an α-level of 0.05, a power of 0.8, and an estimated 10% prevalence level of gingival recession. The remaining statistical analyses were conducted using SAS/STAT 14.01 (SAS Institute Inc., Cary, NC, USA).

Ordinary least square regression models were computed using the various questions as the dependent variable and the presence/absence of fixed retainers in the maxilla or mandible as the exposure variables. Each regression model was adjusted for the following possible confounding variables: age, gender, smoking, use of snuff, number of tooth extractions, duration of orthodontic treatment, and years since debonding.

## 3. Results

Patient demographics for the study population were previously reported by the research group (Table 1) [21].

Seventy-seven percent of the patients experienced no difficulty maintaining oral hygiene, and 13% experienced dental sensitivity (Table 2).

Gingival bleeding was noticed by 69% of patients. Gingival recession and the elongation of teeth were reported by 11% and 10% of patients, respectively. Retainer failure was reported by 59%. Ninety-seven percent was satisfied with orthodontic treatment and treatment result. Having a retainer was not associated with the reduced ability to perform oral hygiene, nor were any confounding factors (Table 3).

Fixed retainers were not related to dental sensitivity (Table 4), nor were any confounding factors.

Smoking was associated with a higher frequency of patient-reported gingival bleeding (*p* < 0.05) (Table 5).

Females more often reported gingival recessions and the elongation of teeth (*p* < 0.05) (Table 6 and Table 7, respectively).

Patients with a long duration of orthodontic treatment more often experienced retainer failure (*p* < 0.05) (Table 8).

Having a retainer in the mandible was associated with reduced satisfaction with orthodontic treatment (*p* < 0.05). No other variables were associated with decreased satisfaction with treatment (Table 9).

Older age was associated with increased reports of contentment with orthodontic treatment results (*p* < 0.05) (Table 10).

## 4. Discussion

The current study explored patient-reported outcome measures on oral health, retainer failure, satisfaction with orthodontic treatment and outcome, and their association with individual indicators in long-term retention patients. The patients’ perspective on oral health has generally received much less attention in the literature compared to the clinicians’ objective outcome measures. Few studies have explored the patients’ perspective on oral health using PROMs, and patient satisfaction has generally received limited coverage in the orthodontic literature. Long-term retention following active orthodontic treatment may influence self-perceived periodontal health and patient satisfaction. Yet, to our best knowledge, no studies have investigated whether self-perceived periodontal health, satisfaction with orthodontic treatment, and satisfaction with orthodontic treatment outcomes become affected in long-term orthodontic retention patients. The present study shows that the presence of a fixed lingual retainer was not associated with patient-reported ability to maintain oral hygiene, periodontal health, need for the replacement of retention, or outcome satisfaction. On the other hand, a fixed lingual retainer in the mandible was associated with patients being less satisfied with the orthodontic treatment itself.

Various clinical signs—including plaque and calculus accumulation, bleeding on probing, gingival recession, and increased periodontal pockets—can indicate damage to periodontal tissues caused by fixed retainers. Plaque accumulation in patients with maxillary bonded retainers has been reported in short- and long-term retention patients. On the other hand, one study showed a decreased presence of plaque three years in retention. Sawhney reported that 12% of patients with a maxillary retainer found it challenging to keep the retainer area clean [22]. In comparison, 17% of patients with a mandibular retainer had difficulty maintaining a clean retainer area. In the current study, having a retainer in the maxilla or mandible was not associated with a reduced ability to perform adequate oral hygiene or with any of the parameters related to oral health (dental sensitivity, gingival bleeding, gingival recession, and tooth elongation). This contrasts the results of Forde et al., who reported that patients preferred removable retainers rather than bonded retainers due to comfort and ability to maintain adequate oral hygiene [23]. Our findings are also in contrast to those reported by Pandis et al., who showed that long-term retention patients have higher calculus accumulation than short-term retention patients [9]. Several studies have illustrated that a subgingival microbiological flora in patients with long-term fixed appliances shifts towards a periodontal/pathogenic character over time [24,25]. In a study evaluating salivary biomarkers in orthodontic patients conducted by Siustis et al., biomarkers in periodontally affected tissues were shown to be significantly reduced after periodontal treatment, emphasizing the need for regular periodontal maintenance during orthodontic treatment [26].

As a retainer in the maxilla or the mandible was not associated with the reduced ability to perform adequate oral hygiene in our study, we speculate that microbiological changes during orthodontic treatment have shifted towards normalization after the termination of orthodontic treatment in this population and that proper hygiene in conjunction with the fixed retention has prevented symptoms of periodontal disease. In a study by Grytten et al., investigating the utilization of dental services in the adult population in Norway, 80% of respondents had been to the dentist during the last year [27]. The Norwegian welfare policy ensures free dental care for children up to the age of 18, and periodontal health is hence under the supervision of the public dental health service/DOT. We therefore assume that the majority of the patients are periodontally healthy.

In our study, females reported gingival recessions more often than men. A gingival recession is described as a displacement of the gingival margin apically from the cementoenamel junction. Although the resulting root exposure can sometimes be associated with increased sensitivity in the dentition [28], we found no gender differences in reports on sensitivity. In a study by Romano et al., it was reported that females were 1.7 times more likely to correctly perceive their gingival condition than men. Our study’s higher frequency of female-reported gingival recessions may be attributed to females’ more significant interest in their health. Conversely, females’ greater interest in their health could also result in an increased frequency of tooth brushing, which is considered a likely etiological factor for gingival recessions [29]. Collectively, the patient-reported outcome measures on periodontal health in this study are in line with the results of a recent systematic review that concluded that fixed retainers are compatible with adequate periodontal health [14].

We previously demonstrated that smoking was associated with gingival bleeding evident upon clinical examination [21]. The current study also showed that patient-reported gingival bleeding was associated with smoking. Although smoking is a well-documented risk factor in periodontitis [30], smoking is considered to be associated with reduced gingival bleeding. We therefore speculate that the increased gingival bleeding in smokers in our study was due to more severe plaque deposits alongside an inferior hygiene regimen in these patients. The use of snuff can cause gingival recessions at the site of placement [31], but this was not supported in our study. Still, the low number of patients that smoked or used snuff in the present study limits the interpretation of these results.

The failure of fixed retainers has a multifactorial origin, and failure rates considerably vary for the different types of retainers. For the most commonly used multi-stranded retainers, the failure rate has been reported as 9–52% [32,33]. The separation between retainer wire and tooth surface, inadequate wire placement or bonding technique, and torque movements of retainer wires are common causes [34]. In the current study, a longer duration of orthodontic treatment was associated with increased fixed retainer failure. A possible explanation is that a more extended period of orthodontic treatment might implicate a more severe malocclusion at baseline. A more complicated treatment course, potentially followed by difficulties obtaining a good occlusion, might increase the risk of mechanical deformation during biting. Treatment difficulty undoubtedly influences the outcome of orthodontic treatment [35] and may affect the failure of fixed retention.

In our study, having a retainer in the mandible was associated with a reduced satisfaction with orthodontic treatment. It is conceivable that the burden of paying extra visits for retainer replacement or repairs could impair the patients’ experience of orthodontic treatment. Yet, in a systematic review, Bondemark et al. argued that there was insufficient evidence to conclude on governing factors of patient satisfaction with the long-term stability of orthodontic treatment [36]. In a study by Sinha et al., it was reported that the most crucial factor that predicted patient satisfaction was the orthodontist’s politeness towards the patient [37]. Other studies have also confirmed the importance of the doctor–patient relationship to ensure patient satisfaction [38]. The latter study found no significant relationship between gender and patient satisfaction, which is in line with our results. Our study also showed that older patients were more content with the result of their orthodontic treatment. This is in contrast to previous studies stating that age is not expected to be a significant factor affecting satisfaction with orthodontic treatment and results [18,37]. Still, there is an inconsistency in the literature regarding the association between dentofacial satisfaction and age [39]. Van Wezel et al. compared the satisfaction and expectations of patients with the results of a study ten years prior, and they reported greater contemporary expectations of orthodontic treatment. Hence, we speculate that the older patients in the present study had fewer expectations of the orthodontic treatment result than the younger patients and therefore reported greater satisfaction with the outcome of treatment.

We detected no relationship between gender and patient satisfaction with orthodontic treatment/treatment results. This is in line with the work of Keles et al., who reported that gender does not predict patient satisfaction [38]. On the other hand, Sheats et al. reported that females were more dissatisfied with their dentition than males after orthodontic treatment [40]. Al-Omiri and Abu Alhaija found that age, gender, and orthodontic treatment need were not associated with patient satisfaction [18]. In contrast to our findings, the latter study also showed that non-extraction patients were more dissatisfied with their dentition. In our study, patients were generally satisfied with orthodontic treatment; only three percent of the patients were dissatisfied.

The use of a questionnaire as a research instrument has inherent limitations due to potential biases. Sources of questionnaire biases can be related to the design of each question, the design of the questionnaire as a whole, and the administration of the questionnaire [41]. For instance, questions can be leading or ambiguous and the interviewer could be non-objective. Hence, to minimize the risk of biases, the questionnaire in the current study was designed and formulated to be as specific as possible. Furthermore, all the participants had finished their orthodontic treatment, and the questionnaire was administered independently of the treating orthodontist. Our study may also have been subject to selection bias because we only included patients that had been treated at the University of Oslo. Thus, caution should be exercised before attempting to generalize our results to a broader population. Finally, our study did not consider the nature of the orthodontic treatment, which therefore may have had unrecognized effects on the patient-reported outcomes.

## 5. Conclusions

In conclusion, long-term orthodontic retention patients reported the satisfactory ability to maintain oral health and are content with orthodontic treatment and outcomes. Fixed retainers alone were not associated with increased difficulties maintaining oral hygiene, reduced self-perceived periodontal health, or less satisfaction with orthodontic treatment outcomes. A fixed retainer in the mandible was associated with decreased satisfaction with orthodontic treatment. In that respect, long-term retention treatment generally appears to be a well-accepted intervention to keep a stable alignment and counteract age-related changes in the anterior dentition. However, additional factors—including gender, the duration of treatment, and age—may be associated with the self-perceived presence of gingival recession and reduced satisfaction with orthodontic treatment.

## Figures and Tables

**Table 1:** Patient demographics [21].

	Mean (Range)	N (%)
Number of patients		211
Retainer in the maxilla		116 (55)
Retainer in the mandible		156 (74)
Retainer in the maxilla and mandible		99 (47)
Females		116 (55)
Smokers		6 (3)
Using snuff		61 (29)
Age in years	20.9 (15–30)	
Number of tooth extractions	1.2 (0–4)	
Duration of orthodontic treatment in months	23.4 (0–72)	
Years since debonding	6.3 (3–10)	

**Table 2:** Patient-reported outcomes in retainer patients (N = 211).

	Percentage
Experience difficulty in maintaining oral hygiene	23
Experience dental sensitivity	13
Experience gingival bleeding	69
Experience gingival recession	11
Experience elongation of teeth	10
Experience retainer failure	59
Content with orthodontic treatment	97
Content with orthodontic treatment result	97

**Table 3:** Predictors of patient-reported oral hygiene performance.

	Maxilla	Mandible
**Independent Variables**		
Retainer in the maxilla	0.05 (0.06)	
Retainer in the mandible		0.04 (0.08)
Age in years	0.01 (0.02)	0.02 (0.02)
Female	0.05 (0.06)	0.05 (0.06)
Smoking	0.10 (0.18)	0.10 (0.18)
Use of snuff	0.19 (0.11)	0.19 (0.11)
Number of tooth extractions	−0.01 (0.02)	−0.01 (0.02)
Duration of orthodontic treatment in months	−0.002 (0.003)	−0.002 (0.003)
Years since debonding	−0.003 (0.02)	−0.0004 (0.02)

Regression coefficients with standard errors in brackets.

**Table 4:** Predictors of patient-reported dental sensitivity.

	Maxilla	Mandible
**Independent Variables**		
Retainer in the maxilla	−0.06 (0.05)	
Retainer in the mandible		−0.03 (0.06)
Age in years	0.02 (0.01)	0.02 (0.01)
Female	0.008 (0.05)	0.006 (0.05)
Smoking	0.01 (0.13)	0.02 (0.13)
Use of snuff	0.02 (0.08)	0.02 (0.08)
Number of tooth extractions	0.0006 (0.01)	0.0004 (0.01)
Duration of orthodontic treatment in months	0.001 (0.003)	0.001 (0.003)
Years since debonding	−0.03 (0.02)	−0.03 (0.02)

Regression coefficients with standard errors in brackets.

**Table 5:** Predictors of patient-reported gingival bleeding.

	Maxilla	Mandible
**Independent Variables**		
Retainer in the maxilla	−0.06 (0.07)	
Retainer in the mandible		−0.03 (0.08)
Age in years	0.02 (0.02)	0.02 (0.02)
Female	0.08 (0.07)	0.07 (0.07)
Smoking	0.38 (0.19) *	0.39 (0.19) *
Use of snuff	0.11 (0.11)	0.10 (0.12)
Number of tooth extractions	−0.02 (0.02)	−0.02 (0.02)
Duration of orthodontic treatment in months	−0.001 (0.004)	−0.001 (0.004)
Years since debonding	−0.04 (0.02)	−0.04 (0.02)

Regression coefficients with standard errors in brackets. * *p* < 0.05.

**Table 6:** Predictors of patient-reported gingival recession.

	Maxilla	Mandible
**Independent Variables**		
Retainer in the maxilla	0.03 (0.04)	
Retainer in the mandible		0.09 (0.05)
Age in years	0.007 (0.01)	0.007 (0.01)
Female	0.11 (0.04) *	0.11 (0.04) *
Smoking	0.20 (0.12)	0.20 (0.11)
Use of snuff	0.04 (0.07)	0.04 (0.07)
Number of tooth extractions	0.02 (0.01)	0.02 (0.01)
Duration of orthodontic treatment in months	−0.001 (0.002)	−0.001 (0.002)
Years since debonding	0.00005 (0.02)	−0.00008 (0.01)

Regression coefficients with standard errors in brackets. * *p* < 0.05.

**Table 7:** Predictors of patient-reported lengthening of teeth as an indicator of gingival recession.

	Maxilla	Mandible
**Independent Variables**		
Retainer in the maxilla	0.04 (0.04)	
Retainer in the mandible		0.06 (0.05)
Age in years	0.008 (0.01)	0.009 (0.01)
Female	0.08 (0.04) *	0.09 (0.04) *
Smoking	0.19 (0.11)	0.19 (0.11)
Use of snuff	0.03 (0.07)	0.03 (0.07)
Number of tooth extractions	0.02 (0.01)	0.02 (0.01)
Duration of orthodontic treatment in months	0.0006 (0.002)	0.0009 (0.002)
Years since debonding	−0.001 (0.01)	−0.003 (0.01)

Regression coefficients with standard errors in brackets. * *p* < 0.05.

**Table 8:** Predictors of patient-reported retainer failure.

	Maxilla	Mandible
**Independent Variables**		
Retainer in the maxilla	0.05 (0.07)	
Retainer in the mandible		−0.14 (0.09)
Age in years	0.01 (0.02)	0.01 (0.02)
Female	−0.03 (0.07)	−0.30 (0.07)
Smoking	−0.09 (0.21)	−0.08 (0.21)
Use of snuff	0.03 (0.13)	0.05 (0.13)
Number of tooth extractions	−0.007 (0.02)	−0.008 (0.02)
Duration of orthodontic treatment in months	0.009 (0.004) *	0.009 (0.004) *
Years since debonding	−0.03 (0.03)	−0.03 (0.02)

Regression coefficients with standard errors in brackets. * *p* < 0.05.

**Table 9:** Predictors of patient-reported orthodontic treatment satisfaction.

	Maxilla	Mandible
**Independent Variables**		
Retainer in the maxilla	−0.04 (0.03)	
Retainer in the mandible		−0.10 (0.04) *
Age in years	0.002 (0.009)	0.0008 (0.009)
Female	−0.006 (0.03)	0.003 (0.003)
Smoking	−0.06 (0.13)	−0.06 (0.13)
Use of snuff	0.05 (0.06)	0.05 (0.06)
Number of tooth extractions	−0.005 (0.008)	−0.004 (0.008)
Duration of orthodontic treatment in months	0.003 (0.002)	−0.003 (0.002)
Years since debonding	−0.004 (0.01)	−0.0005 (0.01)

Regression coefficients with standard errors in brackets. * *p* < 0.05.

**Table 10:** Predictors of patient-reported orthodontic treatment result satisfaction.

	Maxilla	Mandible
**Independent Variables**		
Retainer in the maxilla	−0.01 (0.03)	
Retainer in the mandible		0.04 (0.04)
Age in years	0.02 (0.009) *	0.02 (0.009) *
Female	−0.02 (0.03)	−0.02 (0.03)
Smoking	−0.03 (0.13)	−0.01 (0.13)
Use of snuff	−0.05 (0.06)	−0.06 (0.06)
Number of tooth extractions	−0.003 (0.008)	−0.003 (0.008)
Duration of orthodontic treatment in months	−0.001 (0.002)	−0.001 (0.002)
Years since debonding	−0.02 (0.01)	−0.02 (0.01)

Regression coefficients with standard errors in brackets. * *p* < 0.05.

## Data Availability

Not applicable.
